# *catena*-Poly[[diaqua­rubidium(I)](μ_2_-3-carboxy­pyrazine-2-carboxyl­ato)(μ_2_-pyrazine-2,3-dicarboxylic acid)]

**DOI:** 10.1107/S1600536809002001

**Published:** 2009-01-23

**Authors:** Mustafa Tombul, Kutalmis Guven

**Affiliations:** a Department of Chemistry, Faculty of Art and Science, University of Kırıkkale, Campus, Yahsihan, 71450 Kırıkkale, Turkey; b Department of Physics, Faculty of Art and Science, University of Kirikkale, Campus, Yahsihan, 71450 Kırıkkale, Turkey

## Abstract

The structural unit of the title compound, [Rb(C_6_H_3_N_2_O_4_)(C_6_H_4_N_2_O_4_)(H_2_O)_2_]_*n*_, consists of one rubidium cation, one hydrogen pyrazine-2,3-dicarboxyl­ate anion, one pyrazine-2,3-dicarboxylic acid mol­ecule and two water mol­ecules. This formulation is repeated twice in the asymmetric unit as the rubidium cation lies on an inversion centre. Each anion or acid mol­ecule is linked to two rubidium cations, while the rubidium cation has close contacts to four symmetry-equivalent organic ligands, with two different coordination modes towards this cation. In addition, each rubidium cation is coordinated by two water O atoms, raising the coordination number to eight. One of the carboxyl groups of the acid holds its H atom, which forms a hydrogen bond to a coordinated water mol­ecule. The other carboxyl group is deprotonated in half of the ligands and protonated in the other half, taking part in a strong O—H⋯O hydrogen bond disordered over an inversion centre. The stabil­ization of the crystal structure is further assisted by O—H⋯O and O—H⋯N hydrogen-bonding inter­actions involving the water mol­ecules and carboxyl­ate O atoms.

## Related literature

Pyrazine-2,3-dicarboxylic acid (Takusagawa & Shimada, 1973 ▶) and its dianion (Richard *et al.*, 1973 ▶; Nepveu *et al.*, 1993 ▶) have been used in the construction of multi-dimensional frameworks. A variety of metal–pyrazine-2,3-dicarboxylic acid complexes have been characterized, including the calcium (Ptasiewicz-Bak & Leciejewicz, 1997*a* ▶; Starosta & Leciejewicz, 2005 ▶), magnesium (Ptasiewicz-Bak & Leciejewicz, 1997*b* ▶), sodium (Tombul *et al.*, 2006 ▶), caesium (Tombul *et al.*, 2007 ▶), potassium (Tombul *et al.*, 2008*a* ▶) and lithium (Tombul *et al.*, 2008*b* ▶) complexes. For Rb—N bond lengths, see: Yang *et al.* (2008 ▶); Cametti *et al.* (2005 ▶); Wiesbrock & Schmidbaur (2003 ▶); Shannon (1976 ▶); Devi & Vidyasagar (2000 ▶).
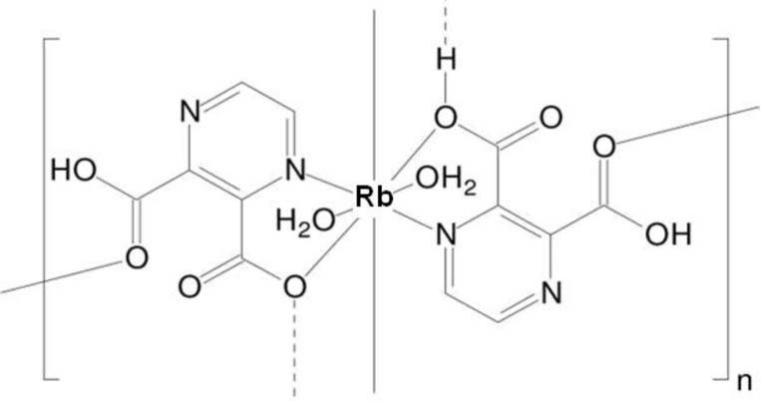


## Experimental

### 

#### Crystal data

[Rb(C_6_H_3_N_2_O_4_)(C_6_H_4_N_2_O_4_)(H_2_O)_2_]
*M* *_r_* = 456.72
Triclinic, 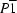

*a* = 7.452 (2) Å
*b* = 7.8640 (15) Å
*c* = 8.3280 (12) Å
α = 69.111 (12)°
β = 81.424 (19)°
γ = 65.322 (18)°
*V* = 414.32 (16) Å^3^
*Z* = 1
Mo *K*α radiation
μ = 3.05 mm^−1^
*T* = 298 (2) K
0.20 × 0.15 × 0.06 mm

#### Data collection

Rigaku AFC-7S diffractometer
Absorption correction: numerical (Clark & Reid, 1995 ▶) *T*_min_ = 0.581, *T*_max_ = 0.838
5329 measured reflections
5057 independent reflections
1604 reflections with *I* > 2σ(*I*)
*R*_int_ = 0.048
3 standard reflections every 150 reflections intensity decay: none

#### Refinement

*R*[*F* ^2^ > 2σ(*F* ^2^)] = 0.053
*wR*(*F* ^2^) = 0.219
*S* = 0.98
5057 reflections
133 parameters
1 restraint
H atoms treated by a mixture of independent and constrained refinement
Δρ_max_ = 1.35 e Å^−3^
Δρ_min_ = −1.91 e Å^−3^

### 

Data collection: *MSC/AFC Diffractometer Control Software* (Molecular Structure Corporation, 1997 ▶); cell refinement: *MSC/AFC Diffractometer Control Software*; data reduction: *TEXSAN for Windows* (Molecular Structure Corporation, 1997 ▶); program(s) used to solve structure: *SHELXS97* (Sheldrick, 2008 ▶); program(s) used to refine structure: *SHELXL97* (Sheldrick, 2008 ▶); molecular graphics: *Mercury* (Macrae *et al.*, 2006 ▶); software used to prepare material for publication: *publCIF* (Westrip, 2009 ▶).

## Supplementary Material

Crystal structure: contains datablocks global, I. DOI: 10.1107/S1600536809002001/hg2450sup1.cif

Structure factors: contains datablocks I. DOI: 10.1107/S1600536809002001/hg2450Isup2.hkl

Additional supplementary materials:  crystallographic information; 3D view; checkCIF report

## Figures and Tables

**Table 1:** Selected geometric parameters (Å, °)

Rb1—O3^i^	2.987 (3)
Rb1—N2^ii^	3.007 (3)
Rb1—O5	3.096 (4)
Rb1—O1	3.137 (3)

Symmetry codes: (i) 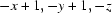; (ii) 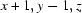.

**Table 2:** Hydrogen-bond geometry (Å, °)

*D*—H⋯*A*	*D*—H	H⋯*A*	*D*⋯*A*	*D*—H⋯*A*
O3—H1⋯O3^iv^	0.93	1.55 (1)	2.468 (3)	170 (1)
O5—H5*A*⋯N1	0.84 (9)	2.07 (9)	2.888 (3)	168 (7)
O5—H5*B*⋯O4^v^	0.82 (6)	1.94 (6)	2.753 (3)	170.39 (5)
O2—H2⋯O5^vi^	0.82	1.79	2.596 (6)	166 (1)

Symmetry codes: (iv) 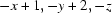; (v) 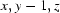; (vi) 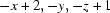.

**Fig. 1.**
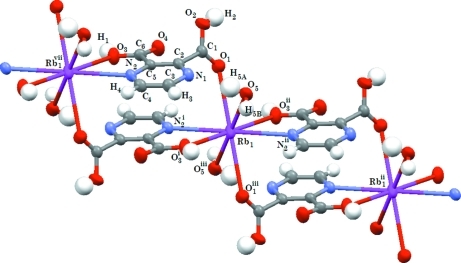
A perspective view diagram of the structure of (I) together with asymmetric units. Displacement ellipsoids are drawn at the 50% probability level. [Symmetry codes: (i) 1 - x, 1 - y, -z, (ii) 1 + x, y - 1, z, (iii) -x + 2, -y, -z, (vii) x - 1, y + 1, z].

**Fig. 2.**
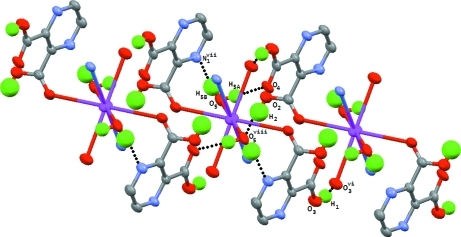
Hydrogen bongd diagram of (I). Dashed lines indicate hydrogen bonds. (H atoms of carbons were ommited for clarity).

## supplementary crystallographic information

### Comment

Pyrazine-2,3-dicarboxylic acid (Takusagawa & Shimada, 1973) and its dianion (Richard *et al.*, 1973; Nepveu *et al.*, 1993) have been reported to be well suited for the construction of multidimensional frameworks (nD, n = 1–3), owing to the presence of two adjacent carboxylate groups (O donor atoms) as substituents on the N-heterocyclic pyrazine ring (N donor atoms). In recent years, a variety of metal-organic compound of pyrazine-2,3-dicarboxylic acid have been characterized crystallographically owing to growing interest in supramolecular chemistry. Examples are including the calcium (Ptasiewicz-Bak & Leciejewicz, 1997*a*; Starosta & Leciejewicz, 2005), magnesium (Ptasiewicz-Bak & Leciejewicz, 1997*b*), sodium (Tombul *et al.*, 2006), caesium (Tombul *et al.*, 2007) and potassium (Tombul *et al.*, 2008*a*) and lithium (Tombul *et al.*, 2008*b*) complexes. Continuation our research on Group I dicarboxylates, we present here the synthesis and crystal structure of the hydrated polymeric rubidium complex, (I), formed with pyrazine-2,3-dicarboxylic acid.

The structural unit of the title compound, (I), contains one rubidium cation, one hydrogen pyrazine-2,3-dicarboxylate anion, one pyrazine-2,3-dicarboxylic acid molecule and two water molecules; this is twice in the asymmetric unit, since the rubidium ion lies on an inversion centre. This compound is isostructural with the corresponding potassium and caesium complexes, which are fully described previously (Tombul *et al.*, 2008*a*; Tombul *et al.*, 2007). Pyrazine-2,3-dicarboxylic acid is, on average, only half deprotonated at one of the carboxylate groups (O3^ii^ and O3^iii^) complete the charge balance of the cation. Taking a larger domain of the crystal structure, the anion or acid molecule is linked to two rubidium cations, while the Rb^+^ cation is surrounded by four organic ligands, two of which are coordinated by employing both N and O atoms and the other two are coordinated solely by O atoms. In addition, each rubidium cation is coordinated by two water molecules, raising a coordination number of eight. The inner coordination sphere accommodates comprises six oxygen atoms (O1, O1^i^, O3^ii^, O3^iii^, O5 and O5^i^), together with two nitrogen atoms (N2^ii^ and N2^iii^). The planes of the carboxylic/carboxylate groups (O1/C1/C2/O2) and (O3/C6/C5/O4) form dihedral angles with the ring (C2/C3/C4/C5/N1/N2) plane of 75.87 (16) and 6.65 (21), respectively. The Rb—O distances are in the range from 2.987 (3) Å to 3.137 (3) Å, which are well within the range reported in the literature for other rubidium complexes (Yang *et al.* 2008; Cametti *et al.*, 2005; Wiesbrock & Schmidbaur, 2003; Shannon, 1976; Devi & Vidyasagar, 2000). Rb—N bond lengths also lie within the normal ranges found for similar bonds in the literature (Yang *et al.* 2008).

In the crystal structure, an asymmetric strong hydrogen bond occurs, linking carboxylate O atoms O—H···O [O···O = 2.468 (3) 2.596 (2) Å respectively]. Atom H1 is involved in this bond and maintains the charge balance within the structure. The water molecules are involved in normal, slightly bent, hydrogen bonds with hydrogen pyrazine-2,3-dicarboxylate (Table 2); the acceptors are carboxylate O atoms and N atoms of the aromatic ring. The polymeric complex is linked in a three-dimensional manner by further numerous intermolecular O—H···O and O—H···N hydrogen bonds (Fig. 2 and Table 2)

### Experimental

Rb~2~CO~3~ (462 mg, 2 mmol) was carefully added to an aqueous solution (20 ml) of pyrazine 2,3-dicarboxylic acid (672 mg, 4 mmol), until no further bubbles formed. The reaction mixture produced a colourless and clear solution which was stirred at 323 K for 200 min., until it solidified. The solid product was then redissolved in water (5 ml) and allowed to stand for three days at ambient temperature, after which transparent fine crystals were harvested.

### Refinement

The H5A atom of water molecule was located in a difference map. The position of H5B atom was obtained from difference map and then its position was refined with riding constraint.The other hydyrogen atoms (H1, H2, H3, H4) were repositioned geometrically. (C—H = 0.93 Å, O—H = 0.93 Å and O—H = 0.82 Å) and U\ĩso\~(H) (in the range 1.2–1.5 times U\~eq\~ of the parent atom).

### Crystal data

**Table:**

[Rb(C_6_H_3_N_2_O_4_)(C_6_H_4_N_2_O_4_)(H_2_O)_2_]	*Z* = 1
*M**_r_* = 456.72	*F*(000) = 228
Triclinic, *P*1	*D*_x_ = 1.830 Mg m^−^^3^
Hall symbol: -P 1	Mo *K*α radiation, λ = 0.71073 Å
*a* = 7.452 (2) Å	Cell parameters from 25 reflections
*b* = 7.8640 (15) Å	θ = 2.6–9.2°
*c* = 8.3280 (12) Å	µ = 3.05 mm^−^^1^
α = 69.111 (12)°	*T* = 298 K
β = 81.424 (19)°	Prism, colourless
γ = 65.322 (18)°	0.20 × 0.15 × 0.06 mm
*V* = 414.32 (16) Å^3^	

### Data collection

**Table:**

Rigaku AFC-7S diffractometer	1604 reflections with *I* > 2σ(*I*)
Radiation source: fine-focus sealed tube	*R*_int_ = 0.048
graphite	θ_max_ = 40.0°, θ_min_ = 2.6°
ω–2θ scans	*h* = −13→13
Absorption correction: numerical (Clark & Reid, 1995)	*k* = −12→13
*T*_min_ = 0.581, *T*_max_ = 0.838	*l* = 0→15
5329 measured reflections	3 standard reflections every 150 reflections
5057 independent reflections	intensity decay: none

### Refinement

**Table:**

Refinement on *F*^2^	Secondary atom site location: difference Fourier map
Least-squares matrix: full	Hydrogen site location: inferred from neighbouring sites
*R*[*F*^2^ > 2σ(*F*^2^)] = 0.053	H atoms treated by a mixture of independent and constrained refinement
*wR*(*F*^2^) = 0.219	*w* = 1/[σ^2^(*F*_o_^2^) + (0.0711*P*)^2^ + 0.5448*P*] where *P* = (*F*_o_^2^ + 2*F*_c_^2^)/3
*S* = 0.98	(Δ/σ)_max_ = 0.002
5057 reflections	Δρ_max_ = 1.35 e Å^−^^3^
133 parameters	Δρ_min_ = −1.91 e Å^−^^3^
1 restraint	Extinction correction: *SHELXL97* (Sheldrick, 2008), Fc^*^=kFc[1+0.001xFc^2^λ^3^/sin(2θ)]^-1/4^
Primary atom site location: structure-invariant direct methods	Extinction coefficient: 0.093 (11)

### Special details

**Table:**

Experimental. [analytical numeric absorption correction using a multifaceted crystal model based on expressions derived (Clark & Reid, 1995)]
Geometry. All e.s.d.'s (except the e.s.d. in the dihedral angle between two l.s. planes) are estimated using the full covariance matrix. The cell e.s.d.'s are taken into account individually in the estimation of e.s.d.'s in distances, angles and torsion angles; correlations between e.s.d.'s in cell parameters are only used when they are defined by crystal symmetry. An approximate (isotropic) treatment of cell e.s.d.'s is used for estimating e.s.d.'s involving l.s. planes.
Refinement. Refinement of *F*^2^ against ALL reflections. The weighted *R*-factor *wR* and goodness of fit *S* are based on *F*^2^, conventional *R*-factors *R* are based on *F*, with *F* set to zero for negative *F*^2^. The threshold expression of *F*^2^ > σ(*F*^2^) is used only for calculating *R*-factors(gt) *etc*. and is not relevant to the choice of reflections for refinement. *R*-factors based on *F*^2^ are statistically about twice as large as those based on *F*, and *R*- factors based on ALL data will be even larger.

### Fractional atomic coordinates and isotropic or equivalent isotropic displacement parameters (Å^2^)

**Table:**

	*x*	*y*	*z*	*U*_iso_*/*U*_eq_	Occ. (<1)
Rb1	1.0000	0.0000	0.0000	0.0474 (3)	
O1	0.9512 (4)	0.1940 (5)	0.2826 (4)	0.0451 (7)	
O2	0.8035 (5)	0.3205 (6)	0.4927 (4)	0.0555 (9)	
H2	0.9157	0.2906	0.5229	0.083*	
O3	0.4264 (4)	0.8765 (4)	0.0673 (4)	0.0472 (7)	
H1	0.4742	0.9762	0.0256	0.071*	0.50
O4	0.7256 (4)	0.6622 (5)	0.1716 (5)	0.0497 (8)	
O5	0.8582 (5)	−0.1900 (5)	0.3618 (5)	0.0520 (8)	
N1	0.5456 (5)	0.1950 (5)	0.3236 (4)	0.0367 (7)	
N2	0.2959 (4)	0.5834 (5)	0.1521 (4)	0.0333 (6)	
C1	0.8065 (5)	0.2816 (5)	0.3520 (5)	0.0335 (7)	
C2	0.6004 (5)	0.3460 (5)	0.2892 (4)	0.0307 (7)	
C3	0.3632 (6)	0.2399 (6)	0.2753 (5)	0.0390 (8)	
H3	0.3190	0.1392	0.2980	0.047*	
C4	0.2388 (6)	0.4346 (6)	0.1918 (5)	0.0372 (8)	
H4	0.1117	0.4612	0.1629	0.045*	
C5	0.4790 (5)	0.5392 (5)	0.1997 (4)	0.0297 (7)	
C6	0.5508 (6)	0.7044 (5)	0.1459 (5)	0.0344 (8)	
H5A	0.761 (12)	−0.087 (12)	0.364 (10)	0.10 (3)*	
H5B	0.816 (8)	−0.243 (8)	0.316 (6)	0.057 (16)*	

### Atomic displacement parameters (Å^2^)

**Table:**

	*U*^11^	*U*^22^	*U*^33^	*U*^12^	*U*^13^	*U*^23^
Rb1	0.0433 (4)	0.0367 (3)	0.0434 (4)	−0.0028 (2)	−0.0063 (2)	−0.0051 (2)
O1	0.0316 (14)	0.0510 (17)	0.0607 (19)	−0.0160 (13)	−0.0025 (13)	−0.0265 (15)
O2	0.0423 (16)	0.079 (2)	0.0460 (17)	−0.0112 (16)	−0.0128 (13)	−0.0312 (17)
O3	0.0395 (15)	0.0302 (13)	0.069 (2)	−0.0180 (12)	−0.0093 (14)	−0.0036 (13)
O4	0.0393 (15)	0.0428 (16)	0.075 (2)	−0.0207 (13)	−0.0172 (14)	−0.0157 (15)
O5	0.0471 (18)	0.0489 (18)	0.066 (2)	−0.0120 (15)	−0.0233 (15)	−0.0253 (16)
N1	0.0360 (16)	0.0293 (14)	0.0426 (17)	−0.0139 (12)	−0.0078 (13)	−0.0053 (12)
N2	0.0290 (14)	0.0350 (15)	0.0375 (16)	−0.0144 (12)	−0.0070 (12)	−0.0088 (12)
C1	0.0304 (17)	0.0321 (17)	0.0395 (19)	−0.0143 (14)	−0.0057 (14)	−0.0089 (14)
C2	0.0299 (16)	0.0340 (17)	0.0304 (17)	−0.0148 (14)	−0.0030 (13)	−0.0093 (13)
C3	0.040 (2)	0.0370 (19)	0.046 (2)	−0.0223 (16)	−0.0045 (16)	−0.0094 (16)
C4	0.0324 (17)	0.043 (2)	0.041 (2)	−0.0219 (16)	−0.0042 (15)	−0.0096 (16)
C5	0.0307 (16)	0.0325 (16)	0.0300 (16)	−0.0147 (13)	−0.0050 (13)	−0.0100 (13)
C6	0.0397 (19)	0.0314 (17)	0.0400 (19)	−0.0200 (15)	−0.0049 (15)	−0.0113 (14)

### Geometric parameters (Å, °)

**Table:**

Rb1—O3^i^	2.987 (3)	O4—C6	1.234 (5)
Rb1—O3^ii^	2.987 (3)	O5—H5A	0.84 (9)
Rb1—N2^ii^	3.007 (3)	O5—H5B	0.82 (6)
Rb1—N2^i^	3.007 (3)	N1—C2	1.336 (5)
Rb1—O5	3.096 (4)	N1—C3	1.340 (5)
Rb1—O5^iii^	3.096 (4)	N2—C4	1.326 (5)
Rb1—O1^iii^	3.137 (3)	N2—C5	1.344 (4)
Rb1—O1	3.137 (3)	N2—Rb1^iv^	3.007 (3)
Rb1—H5B	3.16 (5)	C1—C2	1.513 (5)
O1—C1	1.204 (5)	C2—C5	1.389 (5)
O2—C1	1.307 (5)	C3—C4	1.391 (6)
O2—H2	0.8210	C3—H3	0.9300
O3—C6	1.274 (5)	C4—H4	0.9300
O3—Rb1^iv^	2.987 (3)	C5—C6	1.506 (5)
O3—H1	0.9299		
			
O3^i^—Rb1—O3^ii^	180.00 (14)	O5^iii^—Rb1—H5B	165.0 (3)
O3^i^—Rb1—N2^ii^	126.56 (8)	O1^iii^—Rb1—H5B	104.1 (4)
O3^ii^—Rb1—N2^ii^	53.44 (8)	O1—Rb1—H5B	76.0 (10)
O3^i^—Rb1—N2^i^	53.44 (8)	C1—O1—Rb1	131.4 (2)
O3^ii^—Rb1—N2^i^	126.56 (8)	C1—O2—H2	111.2
N2^ii^—Rb1—N2^i^	180.0	C6—O3—Rb1^iv^	128.1 (2)
O3^i^—Rb1—O5	78.94 (9)	C6—O3—H1	116.0
O3^ii^—Rb1—O5	101.06 (9)	Rb1^iv^—O3—H1	115.9
N2^ii^—Rb1—O5	70.89 (9)	Rb1—O5—H5A	95 (5)
N2^i^—Rb1—O5	109.11 (9)	Rb1—O5—H5B	87 (4)
O3^i^—Rb1—O5^iii^	101.06 (9)	H5A—O5—H5B	104 (6)
O3^ii^—Rb1—O5^iii^	78.94 (9)	C2—N1—C3	116.8 (3)
N2^ii^—Rb1—O5^iii^	109.11 (9)	C4—N2—C5	117.1 (3)
N2^i^—Rb1—O5^iii^	70.89 (9)	C4—N2—Rb1^iv^	119.0 (2)
O5—Rb1—O5^iii^	180.00 (13)	C5—N2—Rb1^iv^	123.5 (2)
O3^i^—Rb1—O1^iii^	81.24 (8)	O1—C1—O2	126.2 (3)
O3^ii^—Rb1—O1^iii^	98.76 (8)	O1—C1—C2	121.8 (3)
N2^ii^—Rb1—O1^iii^	76.07 (8)	O2—C1—C2	111.8 (3)
N2^i^—Rb1—O1^iii^	103.93 (8)	N1—C2—C5	121.9 (3)
O5—Rb1—O1^iii^	118.29 (9)	N1—C2—C1	113.0 (3)
O5^iii^—Rb1—O1^iii^	61.71 (9)	C5—C2—C1	125.1 (3)
O3^i^—Rb1—O1	98.76 (8)	N1—C3—C4	121.2 (3)
O3^ii^—Rb1—O1	81.24 (8)	N1—C3—H3	119.4
N2^ii^—Rb1—O1	103.93 (8)	C4—C3—H3	119.4
N2^i^—Rb1—O1	76.07 (8)	N2—C4—C3	122.0 (3)
O5—Rb1—O1	61.71 (9)	N2—C4—H4	119.0
O5^iii^—Rb1—O1	118.29 (9)	C3—C4—H4	119.0
O1^iii^—Rb1—O1	180.00 (14)	N2—C5—C2	120.9 (3)
O3^i^—Rb1—H5B	70.4 (9)	N2—C5—C6	117.8 (3)
O3^ii^—Rb1—H5B	109.6 (9)	C2—C5—C6	121.2 (3)
N2^ii^—Rb1—H5B	69.3 (10)	O4—C6—O3	125.3 (3)
N2^i^—Rb1—H5B	110.7 (10)	O4—C6—C5	118.4 (3)
O5—Rb1—H5B	15.1 (10)	O3—C6—C5	116.1 (3)
			
O3^i^—Rb1—O1—C1	4.0 (4)	Rb1^iv^—N2—C4—C3	−174.7 (3)
O3^ii^—Rb1—O1—C1	−176.0 (4)	N1—C3—C4—N2	1.8 (7)
N2^ii^—Rb1—O1—C1	−127.2 (4)	C4—N2—C5—C2	−1.2 (5)
N2^i^—Rb1—O1—C1	52.8 (4)	Rb1^iv^—N2—C5—C2	171.6 (3)
O5—Rb1—O1—C1	−68.3 (4)	C4—N2—C5—C6	176.2 (3)
O5^iii^—Rb1—O1—C1	111.7 (4)	Rb1^iv^—N2—C5—C6	−11.0 (4)
Rb1—O1—C1—O2	164.6 (3)	N1—C2—C5—N2	3.7 (6)
Rb1—O1—C1—C2	−10.4 (6)	C1—C2—C5—N2	−178.5 (3)
C3—N1—C2—C5	−3.4 (6)	N1—C2—C5—C6	−173.6 (3)
C3—N1—C2—C1	178.7 (3)	C1—C2—C5—C6	4.2 (6)
O1—C1—C2—N1	72.3 (5)	Rb1^iv^—O3—C6—O4	−179.2 (3)
O2—C1—C2—N1	−103.3 (4)	Rb1^iv^—O3—C6—C5	5.3 (5)
O1—C1—C2—C5	−105.6 (5)	N2—C5—C6—O4	−171.9 (4)
O2—C1—C2—C5	78.8 (5)	C2—C5—C6—O4	5.5 (6)
C2—N1—C3—C4	0.7 (6)	N2—C5—C6—O3	3.9 (5)
C5—N2—C4—C3	−1.5 (6)	C2—C5—C6—O3	−178.7 (4)

Symmetry codes: (i) −*x*+1, −*y*+1, −*z*; (ii) *x*+1, *y*−1, *z*; (iii) −*x*+2, −*y*, −*z*; (iv) *x*−1, *y*+1, *z*.

### Hydrogen-bond geometry (Å, °)

**Table:**

*D*—H···*A*	*D*—H	H···*A*	*D*···*A*	*D*—H···*A*
O3—H1···O3^v^	0.93	1.55 (1)	2.468 (3)	170 (1)
O5—H5A···N1	0.84 (9)	2.07 (9)	2.888 (3)	168 (7)
O5—H5B···O4^vi^	0.82 (6)	1.94 (6)	2.753 (3)	170 (1)
O2—H2···O5^vii^	0.82	1.79	2.596 (6)	166 (1)

Symmetry codes: (v) −*x*+1, −*y*+2, −*z*; (vi) *x*, *y*−1, *z*; (vii) −*x*+2, −*y*, −*z*+1.
